# Procedural learning and school‐age language outcomes in children with and without a history of late talking

**DOI:** 10.1111/1460-6984.12751

**Published:** 2022-06-27

**Authors:** Anna Kautto, Elina Mainela‐Arnold

**Affiliations:** ^1^ Department of Psychology and Speech–Language Pathology University of Turku Turku Finland; ^2^ Department of Speech–Language Pathology University of Toronto Toronto ON Canada

**Keywords:** late talking, language development, procedural learning

## Abstract

**Background:**

‘Late talkers’ (LTs) are toddlers with late language emergence that cannot be explained by other impairments. It is difficult to predict which of these children continue to present long‐term restrictions in language abilities and will later be identified as having a developmental language disorder. Procedural memory weaknesses have been suggested to underlie developmental language disorders, but have not been investigated in LTs.

**Aims:**

We investigated the relationships between aspects of procedural memory and school‐age language abilities in children with and without a history of LT. We hypothesized that children with a history of LT exhibit (1) restrictions in procedural memory when compared with children with typical early development (TED); and (2) a positive association between procedural memory and school‐age language abilities.

**Methods & Procedures:**

We recruited 79 children (7;5–10;5), 43 of whom had a history of LT. Aspects of procedural memory, procedural learning and motor planning were assessed using the serial reaction time and the end‐state comfort tasks. School‐age language abilities were measured using standardized tests.

**Outcomes & Results:**

Counter to expectations, motor planning was not associated with a history of LT or school‐age language abilities, and the children with TED did not show stronger procedural learning as compared with peers with a history of LT. However, weaker school‐age language abilities were associated with weak procedural learning in TED group.

**Conclusions & Implications:**

Factors other than deficits in procedural memory are likely to underlie LT. Procedural learning shows promise as a potential predictor of language development in children that are not identified as LTs.

**What this paper adds:**

**What is already known on the subject**

- Poor procedural learning has been associated with developmental language disorders and suggested to underlie language difficulties. However, to our knowledge, this study is the first to investigate procedural learning and its associations with language outcomes in LTs.

**What this paper adds to existing knowledge**

- Consistent with prior research, we found an association between language abilities and procedural learning in school‐aged children, but found no evidence for poor procedural learning in children with a history of LT. Furthermore, the school‐age language outcomes were only associated with procedural learning in children with no history of LT.

**What are the potential or actual clinical implications of this work?**

- Our findings suggest that factors other than limitations in procedural learning underlie LT. However, procedural learning could be a useful predictor for school‐aged language outcomes in children not identified as LTs.

## INTRODUCTION

Children typically produce their first words at around 12 months. However, some children still produce few words and no word combinations at the age of 2 years. Children with late language emergence, but no other disabilities, are referred to as late talkers (LTs). Most LTs catch up with their peers in language skills by school age. These children are sometimes referred to as late bloomers. However, some children will continue to exhibit persistent language learning difficulties.

In the cases where language difficulties persist past the age of 4, the term developmental language disorder (DLD) is used. Children with DLD can perform below age expectations in different areas of language with no other sensory or environmental factors that could explain the difficulties. The aetiology of DLD is still unknown, but it is known to run in families and has been associated with difficulties in other areas of development, such as subtle motor deficits (Bishop, 2006). DLD has been associated with difficulties in both fine and gross motor skills (for a review, see Sanjeevan et al., 2015).

While we still do not know what causes DLD, several theories about the underlying impairment have been proposed. Although many of the hypothesized underlying deficits such as auditory processing difficulties, and processing capacity limitations (Lum & Conti‐Ramsden, 2013) are domain general in nature, only one theory, the Procedural Deficit Hypothesis (PDH; Ullman & Pierpont, 2005), clearly predicts co‐occurring language and motor deficits in children with DLD. Procedural memory supports learning and storing skills involving patterns and regularities, and is implicit and domain‐general in nature. Skills requiring procedural memory include automatized serial movements, such as buttoning up a shirt, typing or walking. PDH suggests that procedural learning is also responsible for learning rule‐governed aspects of language (Ullman & Pierpont, 2005). According to the PDH, weakness in procedural learning leads to difficulties in acquiring language structures, such as morphosyntax or phonotactics.

Many studies have addressed the PDH. Procedural memory has been measured with different tasks, of which serial reaction time (SRT) tasks are the most used. While converging evidence supports the connection between language disorders and procedural learning (Lum et al., 2012), not all studies have found associations between SRT performance and language impairments (Clark & Lum, 2017; Desmottes et al., 2016; Gabriel et al., 2015). Furthermore, a recent meta‐analysis by West et al. (2021) suggests that extreme group designs in most of the studies may have overestimated the relationship between procedural learning and language abilities. They also criticized the use of a single measure of procedural memory on children with DLD. In the current study, we responded to the criticism by treating school‐age language abilities as a continuum and using two different measures of procedural memory.

Sanjeevan et al. (2018) hypothesized that mixed results from sequence learning tasks in children with DLD might be a result of difficulties in *planning* rather than *learning* the sequences. Motor planning refers to a set of processes defining how a motor goal is achieved (Rosenbaum et al., 1990). The End‐State Comfort (ESC) task provides a simple and effective way to measure motor planning (Rosenbaum et al., 1990). An ESC task may involve, for example, turning around a cylinder‐shaped object, such as a mug. Adults tend to plan the motor sequence such that the end state of the sequence is a comfortable ‘thumb up’ grasp. Young children, however, tend to start the movement sequence with the comfortable grasp resulting in a more awkward ‘thumb down’ position at the end of the sequence. Starting with an uncomfortable grasp to end with a comfortable one is thought to reflect the ability to plan the movement series before starting. As a child develops, the likelihood of ESC grasps increases, which is thought to reflect the development of motor planning (Thibaut & Toussaint, 2010).

Sanjeevan et al. (2018) found that children with DLD were more likely than their peers to use an awkward ‘thumb down’ grasp when it is not warranted and suggested that this behaviour might be indicative of planning deficits in DLD. Interestingly though, this study did not find differences between children with DLD and typical language development (TD) peers in the proportion of ESC grasps on target trials. However, the authors interpreted the results to indicate that children with DLD exhibit motor planning deficits.

Even though the evidence reviewed above suggests children with DLD differ from their typically developing peers in procedural learning, to our knowledge, no published study has evaluated these skills in children with a history of LT.

### LT and DLD

LT is perhaps the best‐known predictor of persistent language difficulties and many studies on predictors for DLD have focused on children identified as LTs. However, if we consider LT as an indicator of a language disorder during the early years, we must recognize the relatively low stability of the developmental trajectories of these skills before school age. According to Vehkavuori et al. (2021), expressive vocabulary at 24 months predicted 16% and receptive language skills 35% of the variation in general language scores at 5;0 years. While both expressive lexicon and receptive language abilities were proven to be significant predictors of language at 5 years of age, unexplained variations remain in both domains.

Children's developmental trajectories in language acquisition often differ (Rudolph & Leonard, 2016). On the one hand, many LTs catch up in language skills before school age and, on the other, all children with persistent language difficulties might not be identified as LTs early on. Zambrana et al. (2014) reported that in a population‐based study, 68.4% of participants with a parental report of language difficulties at 5 years of age were not reported to have language difficulties at the age of 3. Armstrong et al. (2017) reported that 26% of children with parent‐reported age‐appropriate language skills at 2 years of age demonstrated language abilities < –1 SD from the population mean in a standardized test at 10 years of age.

The relationship between LT and DLD has been addressed by different theories. According to categorical theories, LT and DLD are qualitatively different phenomena and have different aetiologies. Research within the categorical framework often focuses on investigating markers that are associated with persistent language disorders. These possible markers can be behavioural (such as grammatical difficulties; e.g., Rice et al., 2008) or biological (such as a specific gene, e.g., Bishop, 2006).

In contrast, dimensional accounts state that LT and DLD are varying degrees of the same phenomenon, suggesting that the mechanisms of impairment underlying DLD and LT are the same, but the severity of the impairment determines whether the child exhibits LT only or persistent difficulties associated with DLD (Rescorla, 2009, 2011). Differences in language skills are a result of variation in skills subserving language. These skills are similar to those that have been suggested to underlie the difficulties present in DLD. According to Rescorla, most LTs who score within the normal range on language tests at school age continue to perform significantly lower than their typically developing peers.

Given that according to the PDH, limitations in procedural memory underlie language deficits in DLD and that according to the dimensional account of LT, LT and DLD share the same underlying deficits varying only in degree, we hypothesized that mild procedural deficits result in LT. We set out to evaluate whether children with and without LT histories differ in two aspects of procedural learning: sequence learning and motor planning, and the extent to which those skills are associated with these children's language abilities at school age.

### Current study

In this study we sought to investigate the associations between procedural memory, early LT status and language skills in school age. We hypothesized that low performance on procedural memory tasks would be associated with (1) a history of LT and (2) low school‐age language abilities, reflecting shared underlying mechanisms. On the SRT task, we expected smaller effects of sequence learning, and on the ESC task fewer ESC grasps on target trials to be associated with (1) a history of LT and (2) low school‐age language abilities.

## METHODS

### Participants

This study was conducted as a part of the Southwestern Birth Cohort study (Lagström et al., 2012). It included a total of 9936 children, 1827 of whom participated in follow‐up studies. The cohort study and the current study were approved by the ethics committee of the Hospital District of Southwest Finland. The participants in this study are the same as in Kautto et al. (2021).

Invitation letters were sent to families with children who could be identified as LTs at ages 24 or 36 months during earlier time points in the cohort study. Due to missing data points associated with the cohort study, the number of LT children does not reflect the prevalence of LT in the population that participated in the follow‐up studies. We used the following criteria in defining LT: (1) performance 1.25 SD or more below age expectations on (a) the MacArthur–Bates Communicative Development Inventory, which assesses expressive vocabulary and early word combinations (Fenson et al., 2007; Finnish version Lyytinen, 2000) at 24 months of age (*n* = 19); (b) the Fox Language Inventory (Korpilahti & Eilomaa, 2002), a screening method assessing expressive and receptive language on word and sentence levels, carried out by a clinical nurse at 36 months (*n* = 21); and (c) the Renfrew Word Finding Vocabulary Test at 36 months of age (*n* = 7; see Korpilahti et al., 2016a, 2016b, for details); or (2) speech–language service delivery at 2 or 3 years of age according to parent report (*n* = 17). All criteria could not be used for every participant due to missing data points associated with the cohort study. Consequently, we supplemented the usual LT criteria, limitations in early expressive vocabulary and combining words (1a; Fisher, 2017) with Finnish screening instruments assessing expressive and receptive word and sentence level skills (1b, c). The history of LT could be confirmed for 38 participants using standardized measures (criterion 1a–c). Only three participants were included solely based on criterion 2, early language service delivery. For these children, early speech–language service delivery and LT history were also confirmed by a speech–language pathologist. We also recruited children with no history of LT. These children were required to exhibit (1) performance between –1 and +1 SD on the MacArthur–Bates Communicative Development Inventory at 24 months^1^; and (2) no known history of LT or speech–language intervention any time prior to participating in the current study according to parent report.

The recruitment resulted in a total of 79 children (ages 7;5–10;5) participating in the current study. All participants were required to exhibit (1) normal hearing based upon an audiometry screening at 20 dB HL (1, 2 and 4 kHz);^2^ (2) Finnish spoken as the home language; and (3) no frank emotional, behavioural, motor, intellectual or neurological disability based on parent reports. We also excluded all children whose performance reasoning index (PRI) was < 70 as measured by the Finnish version of the Wechsler Intelligence Scale for Children (WISC‐IV; Wechsler, 2003). Two children were excluded because of a PRI below the cut‐off point, resulting in 77 participants (41 LT, 36 typical early development—TED).

### LT status and school‐aged language abilities

Children's language abilities were assessed using the NEPSY‐II (Developmental Neuropsychological Assessment; Korkman et al., 2007) Narrative Memory and Comprehension of Instructions subtests, and the WISC‐IV Vocabulary subtest at 7–10 years of age. We computed individual Language Index scores as a mean of standard scores on these three subtests, based on Finnish norming samples. Thus, the Language Index scores had a mean of 10 and a SD of 3. Differing from earlier studies on procedural learning and language abilities (for a review, see West et al., 2021), we used the Language Index as a continuous measure instead of a dichotomous measure such as typical development/DLD to account for the full variability of school‐age language abilities. The Language Index was lower in the LT group (*M* = 8.19, SD = 4.02) as compared with the TED group (*M* = 10.06, SD = 5.17), *t*(73.63) = 3.03, *p* = 0.003. Even though a continuous measure of Language Index was used in statistical models, in Table 1 we divided the participants into subgroups of children performing at and below age expectations in standardized language measures (TD and DLD) at school age to describe the participants. A cut‐off of –1.25 SD from the population mean was used. A total of 11 participants from the LT group (26.8%) and four participants from the TED group (11.1%) in our sample had school‐age language abilities below age expectations. Late bloomers (*M* = 9.27, SD = 2.31) as a group performed somewhat lower than children in TED (*M* = 10.62, SD = 2.31) group, *t*(59.76) = 2.29, *p* = 0.025. School‐age language statuses in the LT group suggested that our LT group was very similar to LT groups in previous studies (for a review, see Rescorla, 2011) even though we used local instruments to supplement the more common LT criteria.

**TABLE 1:** Demographic information and performance on standardised tests

	**Late talkers (*N* = 41)**			**Typical early development (*N* = 36)**			
	**LB (*N* = 30)**	**DLD^a^ (*N* = 11)**	**Total LT**	**TD (*N* = 32)**	**DLD^a^ (*N* = 4)**	**Total TED**	***p*‐value^b^**
*Age (months)*							0.231
Mean (SD)	110.77 (9.83)	106.91 (11.07)	109.73 (10.18)	106.50 (6.98)	104.75 (11.15)	106.31 (7.35)	
Range	90–125	89–122	89–125	97–122	93–117	93–122	
*PRI* ^c^							0.018
Mean (SD)	103.13 (17.05)	92.91 (13.72)	100.39 (16.70)	109.31 (17.68)	89.50 (18.34)	107.11 (18.59)	
Range	73–131	71–111	71–131	73–140	71–111	71–140	
*SES* ^d^							0.399
1 low	7 (23.3%)	4 (36.4%)	11 (26.8%)	8 (25.0%)	1 (25.0%)	9 (25.0%)	
2 medium	16 (53.3%)	4 (36.4%)	20 (48.8%)	9 (28.1%)	1 (25.0%)	10 (27.8%)	
3 high	7 (23.3%)	3 (27.3%)	10 (24.4%)	15 (46.9%)	2 (50.0%)	17 (47.2%)	
*Language Index* ^e^							< 0.001
Mean (SD)	9.27 (2.31)	5.24 (0.78)	8.19 (2.70)	10.62 (2.31)	5.58 (.50)	10.06 (2.71)	
Range	6.33–13.67	3.67–6.00	3.67–13.67	6.33–14.67	5.00–6.00	5.00–14.67	
*Comprehension of instructions* ^f^							< 0.001
Mean (SD)	9.97 (2.50)	7.55 (3.11)	9.32 (2.85)	11.91 (2.73)	5.50 (4.20)	11.19 (3.50)	
Range	3–15	3–15	3–15	2–15	1–10	1–15	
*Narrative memory* ^g^							< 0.001
Mean (SD)	7.53 (4.44)	3.55 (2.42)	6.46 (4.35)	9.22 (3.93)	4.25 (2.22)	8.67 (4.07)	
Range	1–15	1–8	1–15	1–15	2–7	1–15	
*Vocabulary* ^h^							< 0.001
Mean (SD)	10.30 (3.19)	4.64 (2.50)	8.78 (3.92)	10.72 (3.09)	7.00 (4.55)	10.31 (3.41)	
Range	4–16	1–8	1–16	5–18	1–12	1–18	

*Note*: LB, late bloomers; LT, late talkers; TD, typical language development.

^a^Developmental language disorder (DLD) status is defined as performance in language tests < 1.25 SD from the population mean.

^b^Analysis of variance (ANOVA) between the four groups (LB, LT‐DLD, TD, TED‐DLD), chi‐square goodness of fit test for socio‐economic status (SES).

^c^Performance reasoning index measured by WISC‐IV.

^d^Maternal education level on a scale of 1–3.

^e^Mean of three subtests (vocabulary, comprehension of instructions and narrative memory).

^f^NEPSY‐II Comprehension of Instructions subtest.

^g^NEPSY‐II Narrative Memory subtest.

^h^WISC‐IV Vocabulary subtest.

The LT and TED groups did not significantly differ in age, LT: *M* = 110 months, SD = 4.58 months, TED: *M* = 106 months, SD = 5.17 months, *t*(72.43) = –1.71, *p* = 0.092, socio‐economic status (SES) measured by maternal education level, LT: *M* = 1.92, SD = 1.00, TED: *M* = 2.22, SD = 0.45, *t*(69.97) = 1.38, *p* = 0.173, or IQ measured by PRI, LT: *M* = 100.39, SD = 13.75, TED: *M* = 107.11, SD = 24.83, *t*(70.98) = 1.66, *p* = 0.101.

### Serial reaction time (SRT) task

**FIGURE 1:**
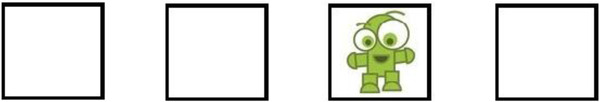
Schematic representation of the serial response time (SRT) task. The creature appears in one box at a time. The child is asked to press the corresponding button. The next target appears after response. After the practice phase (eight trials with feedback), follow pattern phases 1 and 2 (100 trials with order 1‐3‐2‐4‐4‐2‐3‐4‐2‐4 repeating) and a random phase with 100 trials in random order. There is a small break after each phase [Colour figure can be viewed at wileyonlinelibrary.com]

We employed an SRT task with visual stimuli to examine sequence learning (Nissen & Bullemer, 1987; Tomblin et al., 2007). The SRT task was programmed using E‐Prime software (version 2.0.10.356; Psychology Software Tools, Pittsburgh, PA, USA). A schematic description of the task is presented in Figure 1. The task had four different locations (empty boxes) on the screen and a creature appeared in one of the boxes. Children were asked to press the button corresponding to the location of the stimulus as quickly and accurately as possible. The task was self‐paced, meaning that the next stimulus did not appear before the participant pressed one of the buttons. We chose to use a self‐paced task to avoid confusion and frustration which would have arisen from stimuli changing quicker than child could keep up with. The task consisted of a practice procedure (eight trials) and three experimental procedure phases. After the practice procedure, the participant received feedback about the response correctness and latency, to make sure they had understood the instructions. The first two phases of the task were pattern phases in which stimulus locations followed a specific order (1‐3‐2‐4‐4‐2‐3‐4‐2‐4), repeating 10 times in each pattern phase. After the pattern phases, a phase with random stimulus location order followed. Each phase consisted of 100 trials.

The task was presented using a laptop computer and a response box with four buttons to record response times (RTs). Children were advised to use both hands in responding. Participants were seated approximately 60 cm from the computer screen and the stimuli were presented in 13.96° visual angle (all four boxes, single box = 2.38°). The phases were presented in the same order (pattern–pattern–random) for each participant. The length of each phase was 100 trials, and the participant had a possibility to take a small break between the phases.

### End‐State Comfort (ESC) task

To measure motor planning, we employed a modified version of the ESC task (Rosenbaum et al., 1990; Sanjeevan et al., 2018). The task requires the participant to move a dowel from its initial position to another so that it has to be rotated 180° (target trials) or moved without rotation (control trials).

The materials in this task consisted of a board with three plastic cups attached to it. The cups were labelled ‘1’, ‘home’ and ‘2’ from left to right. The dowel used in the task was approximately 23 cm in length and 5 cm in diameter. One end of the dowel was coloured green and the other black.

The dowel was placed in the ‘home’ cup at the beginning of the task, and the participant was instructed to move the dowel to either the left or right cup. The task had two conditions. In the experimental condition (target trials), the dowel needed to be rotated 180° and placed in one of the cups with the black end facing down. In the control condition, the participant was asked to move the dowel from the centre cup to one of the cups on the sides the green end facing down, thereby not requiring the same degree of motor planning as the experimental condition. The dowel was returned to its initial position after each repetition. Instructions were given orally. The task consisted of 16 trials, eight target trials and eight control trials, and in both conditions instructions in each direction were given. Hence, there were four types of instructions in this task (‘Place the [black/green] end of the dowel in cup [one/two].’). The instructions were presented to each participant in a randomised order and were repeated if the participant did not place the correct end of the dowel into the correct cup on the first try. Overall, no participant had difficulty in understanding the instructions.

### Data analyses

R software (R Core Team, 2019) with packages *dplyr* (Wickham et al., 2019) for data manipulation, *lme4* (Bates et al., 2015) for linear models, *hypr* (Rabe et al., 2020) for hypothesis‐based contrast coding and performance (Lüdecke et al., 2020) for assessing model assumptions were used in data analyses, and *sjPlot* (Lüdecke, 2019), *ggeffects* (Lüdecke, 2018) and *ggplot2* (Wickham, 2016) in tables and figures. Analysis scripts are available in supporting information material.

#### SRT task

We modelled RT data from the SRT task. The whole RT distributions for each participant were analysed to take individual variation in RTs into account. One child with a history of LT was excluded from the SRT analyses due to equipment failure. Trials with RTs > 2 SD longer or shorter from each participant's mean RT and trials with < 100 ms RT were excluded. Trials with incorrect response were also excluded from the RT model, resulting in 20,047 trials from 76 participants altogether. Mean RT was 630 ms in TED group (SD = 228 ms) and 590 ms (SD = 211 ms) in LT group, which suggested that our choice of an analysis that takes RT distributions for each participant into account was well justified.

We modelled RTs as a function of LT, Language Index, trial (in blocks of 25), and all two‐ and three‐level interactions of these predictors. RTs were log‐transformed to allow fitting a linear model with a normality assumption. The Language Index was centred at the sample mean. LT and block were categorical measures, LT with two levels (TED/LT), TED as the reference level and block with 12 levels of 25‐trial blocks. Each phase of the 100 trials (pattern 1, pattern 2 and random) consisted of four blocks of 25 trials. We were interested in two effects reflecting procedural learning: RT decrease during pattern phases, ‘the decrease effect’ and RT increase from the end of the pattern phases to the random phase, ‘the task effect’. To model these effects, we employed hypothesis‐based contrast coding for block variable. According to these effects of interest, contrasts were set between the first and the last pattern phase block (blocks 1 and 8, ‘the decrease effect’), and between the last pattern phase block and the random phase blocks (blocks 8 and 9–12, ‘the task effect’). For detailed information about hypothesis‐driven contrast coding, see Rabe et al. (2020). Participant intercept was used as random factor to account for individual variation in RTs.

Our interests were the two predefined contrasts reflecting pattern learning: the decrease effect and the task effect. We hypothesized these effects to be modulated by LT history and school‐age language abilities so that (1) we would observe smaller decrease and task effects in LT than in TED children, suggesting that LT would be associated with weak pattern learning skills; and (2) that children with stronger school‐aged language abilities would show greater decrease and task effects as compared with children with weaker language abilities, indicating that pattern learning is related to language skills.

#### ESC task

Children's grasp types on the dowel task were coded as either a thumb‐up or thumb‐down grasp at the end of the movement. To ensure the reliability, the judgments were scored by a second person. The two coders agreed on the grasps used in all trials.

We analysed grasp types using a generalized linear mixed model employing a binomial distribution, with response type (comfortable or awkward grasp) as a dependent variable, modelled as a function of the target type (target or control), LT, Language Index and their two‐level interactions. Participant intercept was used as a random factor to account for individual variations in the use of grasp types. We hypothesized to observe fewer ESC grasps in the target trials in participants with a low Language Index or a history of LT.

Based on research on children with DLD (Sanjeevan et al., 2018), we also hypothesized to observe a tendency to use unnecessarily awkward grasps in control trials in children with low school‐age language abilities. However, in the control trials, 97.4% of all the grasps were comfortable (TED = 97.3%, LT = 97.4%). Because of this ceiling effect we did not perform statistical analyses to compare the number of unnecessarily awkward grasps between the groups.

## RESULTS

### SRT task

A model summary for SRT performance is presented on the left side in Table 2.

**TABLE 2:** Model summaries for serial reaction time (SRT) task performance

	**log (RT)**				**log (corrected RT)**			
**Predictors**	**Estimate**	**95% CI**	***t*‐value**	***p***	**Estimate**	**95% CI**	***t*‐value**	***p***
(Intercept)	6.42	6.36, 6.48	213.26	**< 0.001**	6.29	6.22, 6.36	181.20	**< 0.001**
Decrease	−0.11	−0.14, –0.08	−7.35	**< 0.001**	−0.33	−0.37, –0.30	−18.58	**< 0.001**
Task effect^a^	0.05	0.02, –0.07	3.87	**< 0.001**	−0.07	−0.09, –0.04	−4.63	**< 0.001**
Language Index	−0.11	−0.17, –0.05	−3.76	**< 0.001**	−0.13	−0.20, –0.06	−–3.77	**< 0.001**
Group [LT]	−0.11	−0.19, –0.03	−2.70	**0.007**	−0.13	−0.22, –0.03	−2.69	**0.007**
Decrease * Language Index	−0.04	−0.07, –0.01	−2.58	**0.010**	−0.08	−0.12, –0.05	−4.68	**< 0.001**
Task effect * Language Index	−0.04	−0.06, –0.01	−2.95	**0.003**	−0.06	−0.09, –0.04	−4.52	**< 0.001**
Decrease * Group [LT]	−0.02	−0.06, –0.02	−1.04	0.298	−0.05	−0.10, –0.00	−1.94	0.053
Task effect * Group [LT]	−0.02	−0.06, –0.01	−1.40	0.160	−0.04	−0.08, –0.00	−1.99	**0.047**
Language Index * Group [LT]	0.08	−0.00, –0.16	1.87	0.062	0.09	−0.00, –0.18	1.89	0.059
(Decrease * Language Index) * Group [LT]	0.03	−0.02, –0.07	1.25	0.210	0.06	0.01, –0.11	2.55	**0.011**
(Task effect * Language Index) * Group [LT]	0.06	0.02, –0.09	3.24	**0.001**	0.08	0.04, –0.12	4.03	**< 0.001**

**Random effects**		
*σ* ^2^	0.09	0.11
*τ*_00_	0.03_id_	0.04_id_
ICC	0.25	0.25
*N*	76_id_	76_id_
Observations	20,047	20,047
Marginal *R* ^2^/conditional *R* ^2^	0.071/0.305	0.095/0.320

*Note*: ^a^Due to the reference level used in the models (last trial block of the pattern phases), positive estimates for the Task effect reflect decrease and negative estimates increase in response times (RTs) from the end of the pattern phases to the beginning of the random phase.

Model with uncorrected RTs (left) and with sustained attention corrected RTs (right).

#### Decrease effect

To verify that the task functioned as expected, we first examined the main effect of RT decrease during pattern phases. This effect (‘Decrease’ in Table 2), reflecting pattern learning, was significant as expected. Consistent with our hypothesis, children with better language abilities exhibited larger RT decrease than peers with weaker abilities, indicating better procedural learning (‘Decrease × Language Index’). Contrary to our hypothesis, TED and LT children did not differ in the magnitude of RT decrease (‘Decrease × Group’). The association between decrease effect and language status (‘Decrease × Language Index × Group’) was observed in TED but not LT participants. However, this interaction was only significant in the model with corrected RTs.

#### Task effect

We observed a RT decrease—not increase—from the last pattern phase to the random phase (‘Task effect’ on the left side of Table 2). This suggests that the SRT task did not function as expected. Our best explanation for this observation was that the rest between the phases resets the effect of sustained attention, at least partly, and that an effect of sustained attention also partly masked the procedural learning during the task (to model this, see the additional supporting information). Maintaining an optimal level of attention to perform a task, especially a long one, is demanding and experimental tasks designed to measure other aspects of cognition often also contain a component of sustained attention (Im‐Bolter et al., 2006). The effect of sustained attention can be observed in the overall increase in RTs during a task. To account for this, we performed a correction for the RTs by extracting a constant reflecting estimated effect of sustained attention decrease during the phases.

**FIGURE 2:**
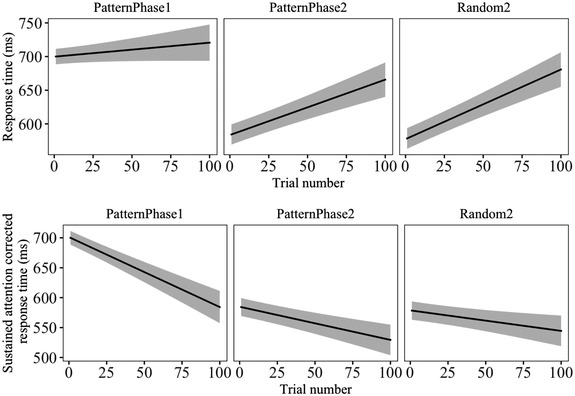
Response times in the serial response time (SRT) task before (above) and after (below) sustained attention correction

The modelled RT value in the beginning of the second pattern phase was 136 ms shorter than in the end of the first pattern phase (Figure 2, upper row). Since the children took a short break between the first and second pattern phases, which were otherwise identical, we assumed that this difference would best represent effects of sustained attention, and used it to model a correction. The RT correction procedure is described in detail in the additional supporting information. We then modelled corrected RTs as a function of LT, Language Index, trial, and all two‐ and three‐level interactions of these predictors, following the same procedure as in our original SRT model. A model summary for the corrected RTs is presented on the right side of Table 2. The model with corrected values shows an RT increase from pattern phases to the random phase (‘Task effect’), indicating that after accounting for sustained attention, the SRT task functioned as expected.

**FIGURE 3:**
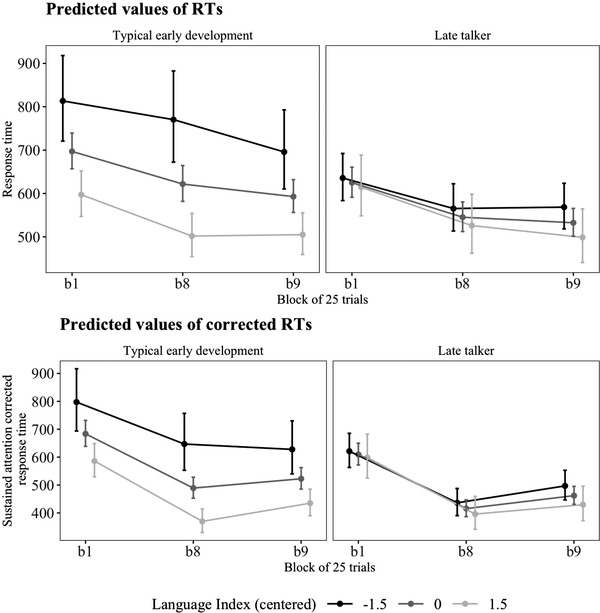
Serial response time (SRT) task performance as a function of block, late talking and Language Index. (upper) Model with uncorrected response times (RTs); and (lower) model with sustained attention corrected RTs. b1 is the first trial block of the pattern phase 1; b8 is the last trial block of the pattern phase 2; and b9 is the random phase. Task effect can be observed in the slope from b8 to b9 and the decrease effect from b1 to b8

All main effects and interactions of the models with corrected and uncorrected RTs are reported in Table 2 and shown in Figure 3. As hypothesized, the children with stronger language abilities showed a larger increase from the pattern phases to the random phase as compared with peers with weaker language abilities (‘Task effect × Language Index’). Contrary to our hypothesis, children with TED and LT did not differ in the task effect as expected (‘Task effect × Group’). The model with corrected values even suggested slightly more pronounced task effect in children with LT as compared with TED. Surprisingly, the relationship of the task effect and language abilities was modulated by LT (‘Task effect × Language Index × Group’). This interaction can be observed in Figure 3. In children with TED, a larger task effect was associated with stronger language abilities. However, in children with LT, task effect was similar in children with varying language abilities.

### End state comfort task

A model summary for the ESC task performance is presented in Table 3. The participants used comfortable end‐state grasps more often in control trials than in target trials (‘Trial’ in Table 3), indicating that the task functioned as intended. Contrary to both our hypotheses that children with a history of LT exhibit poor motor planning when compared with peers and that motor planning predicts school‐age language abilities, the use of ESC grasps in the target trials was not modulated by the history of LT (‘Trial × Late talking’) or Language Index (Trial × Language Index’).

**TABLE 3:** Model summary for ESC task performance

	**ESC responses**			
**Predictors**	**Odds ratios**	**95% CI**	***t*‐value**	***p***
(Intercept)	63.96	25.46–160.71	8.85	**< 0.001**
Trial type [Target]	0.01	0.00–0.02	−10.64	**< 0.001**
Language Index (centred)	0.92	0.41–2.07	−0.20	0.843
Late talking	2.29	0.55–9.52	1.14	0.256
Trial type [Target]* Language Index (centred)	0.90	0.46–1.76	−0.31	0.755
Trial type [Target]* Late talking	0.28	0.07–1.10	−1.82	0.069
Language Index (centred)* Late talking	1.41	0.64–3.09	0.86	0.392

**Random effects**	
*σ* ^2^	3.29
*τ*_00id_	2.05
ICC	0.38
*N*_id_	77
Observations	1232
Marginal *R* ^2^/conditional r^2^	0.567/0.733

## DISCUSSION

In this study we investigated the relationships between procedural memory, school‐age language status and history of LT. We hypothesized that deficits in aspects of procedural memory, procedural learning measured by SRT task and motor planning measured by the ESC task, underlie both LT and later poor school‐age language abilities.

### Sequence learning

The findings did not fully support our hypotheses about the role of procedural learning in language learning. Children with LT and TED did not differ in sequence learning. Neither RT decrease during the pattern phases nor the task effect (RT increase from pattern phases to random phase) indicated weaker performance in children with LT than in TED peers, suggesting that deficits in procedural learning, as measured by SRT task, do not underlie LT.

Contrary to our hypothesis that children with TED would outperform LT children on the SRT task, the LT group showed similar RT decrease effect and even subtly larger task effect than TED peers which indicates that pattern learning is not weaker in children with LT than in children with TED. Based on these findings we suggest that factors above and beyond procedural learning explain LT. Since the language abilities were lower in the LT group as compared with the TED group, we would expect these children also to have a poorer performance in the SRT task if language abilities were modulated by procedural learning.

However, as hypothesized, we did observe a positive relationship between school‐age language abilities and procedural learning as indicated by a smaller SRT task effect and decrease effect in children with poor school‐age language abilities. Unexpectedly, the significant relationship was observed only in children with TED, not in LTs. The positive relationship between school‐age language abilities and procedural learning is in line with previous research supporting the PDH (e.g., Tomblin et al., 2007; Ullman & Pierpont, 2005).

Why did we observe an association between procedural learning and language abilities in children with TED but not LT? We cannot completely rule out the possibility that at least some of the LTs—perhaps those with the persistent language difficulties—have had difficulties in procedural learning during the early years of development, but these difficulties are no longer observable at school age. It is also possible that children with procedural learning difficulties are not LTs to begin with but exhibit a declining language trajectory characterized by language learning difficulties that become apparent only later at school age. According to the procedural learning deficit hypothesis, vocabulary learning relies on declarative more than procedural memory. Given that much of early language development is learning new words, it is reasonable to suggest that this type of deficit does not lead to LT in toddlers. Children with such developmental language trajectories have been described (Armstrong et al., 2017; Rudolph & Leonard, 2016; Zambrana et al., 2014). Future studies should investigate procedural learning in toddler‐aged children with and without LT and combine these results on later language outcomes.

When comparing our results with the findings from other studies investigating the relationship between procedural learning measured by an SRT task and language abilities, it is important to consider that our SRT experiment and analysis procedure differed from earlier studies by three central aspects. First, while most of the earlier studies report a design comparing a group of participants with a language disorder to a control group (for a review, see West et al., 2021), we chose to employ a correlational design measuring individual variation of school‐age language skills as a continuum. While findings from experimental group designs and correlational designs representing full variation in an ability should result in converging evidence, there are also pitfalls associated with using extreme group designs, such as the risk of an inflated effect‐size estimate (Fisher et al., 2020). These pitfalls have been suggested to be one central weakness in earlier studies of procedural learning and language (West et al., 2021). Importantly, we observed an association between procedural learning and school‐aged language abilities without comparing extreme groups.

Second, while most of the earlier language development or DLD studies employing SRT task have analysed RT changes at the phase level, aggregating the data to phase means, we chose to model RT changes also within each phase to fully represent RT decreases during the phases. Analysing RT changes only between and not within the phases could have resulted in modest effect sizes reported in previous studies. In their meta‐analysis, West et al. (2021) pointed out that the effect sizes in studies on procedural learning and language abilities are not large enough to suggest that procedural learning is an important causal risk factor for DLD. One reason for the resulting small between‐subjects differences could be that previous studies did not utilise the full potential of the tasks used to measure procedural learning effects at an individual level, which our modelling did achieve.

We chose to employ a sustained attention correction because without such an approach our results would have reflected the demands the SRT task poses on attention rather than procedural learning. This correction was the third central difference between our study and earlier studies on procedural learning and language abilities. The approach was based on the observation that RTs were notably shorter in the beginning of the second pattern phase than at the end of the first pattern phase. As the correction was similar across all participants, despite individual factors, it did not artificially create between‐subject differences. Without a correction like this, most earlier studies have ended up considering RTs only as means of each phase since they failed to account for the variability associated with sustained attention.

### Motor planning

We also examined motor planning abilities in LT and TED children with varying school‐age language outcomes using the ESC task. We hypothesised that both LT and weak school‐age language abilities would be associated with motor planning difficulties reflected by fewer end‐state grasps in the target trials. Furthermore, given that unnecessary awkward grasps in the control trials were associated with low language abilities in Sanjeevan et al. (2018), perhaps reflecting domain‐general planning deficits associated with DLD, we hypothesised to observe motor planning difficulties in the form of unnecessarily awkward grasps in control trials of the ESC task in children with LT and low language abilities.

The results did not support our hypotheses. The LT and TED groups did not differ in end‐state grasps used in the target trials, nor did the children in the two groups use unnecessarily awkward grasps, suggesting no differences in motor planning between the groups. Furthermore, performance on the ESC task was not associated with school‐age language abilities, providing no evidence for a relationship between language ability and motor planning.

This result was partly in line with earlier research findings on children with DLD (Sanjeevan et al., 2018). In their study, children with DLD did not differ from peers in the use of ESC grasps in target trials requiring turning the dowel. However, differing from the results in Sanjeevan et al., in our study, children with weak language skills did not show a tendency to use unnecessarily awkward grasps in the task. One possible explanation for the difference between this study and Sanjeevan et al. (2018) was that our study included relatively few children clinically identified as having DLD. Sanjeevan et al. recruited school‐aged children with clinically identified language disorders. This yielded children with more severe and persistent language difficulties. However, our study group was representative of LTs.

Another possible explanation for not replicating the findings on perseverative behaviour is the difference in ESC task structure between our study and that by Sanjeevan et al. In our task, we did not control for the relative order of target trials and control trials which resulted in our task having fewer control trials following immediately after target trials. Because of this, we may have had too few trials to properly address the likelihood of perseveration.

However, while acknowledging the differences between the studies, we found no evidence of motor planning difficulties associated with LT or language abilities. Combined with our observations in SRT task, our findings suggest that difficulties in procedural learning associated with weak language skills are not explained by simple planning difficulties, as suggested by Sanjeevan et al. Despite the weaknesses of our ESC task structure for observing perseveration, we are confident that motor planning difficulties that would have seriously affected the SRT results would be so robust that they would also likely have been observed in our ESC task.

### Summary

Children with a history of LT did not show procedural learning difficulties as compared with TD peers. Thus, contrary to our hypothesis, procedural learning does not seem to be associated with a history of LT. However, procedural learning measured by SRT task performance was associated with the school‐age language abilities in children with TED. In our sample, we found a relationship between procedural learning, reflected by sequence learning, and language skills. This relationship was only observed in children with a history of TED, not in LTs. Furthermore, the relationship was present only for procedural learning, not for motor planning, suggesting that not all aspects of procedural memory are equally important for language learning.

### CLINICAL IMPLICATIONS

In the light of our findings, it seems likely that procedural *learning* skills in particular are important for language acquisition, but there are also other important factors affecting language acquisition. Procedural learning does not show promise as a predictor of language outcomes in LTs. Most children with persistent language difficulties share a history of LT. However, it is also important to note that not all children with persistent language difficulties are identified as LTs (Armstrong et al., 2017; Rudolph & Leonard, 2016; Zambrana et al., 2014). Procedural learning could be used in predicting language outcomes in children not identified as LTs. These children are at risk of not receiving early intervention because their language impairments might not be identified before school age. Therefore, our results provide preliminary support for the possibility that procedural learning is a useful predictor of later language difficulties in children without early recognised language learning difficulties. If we manage to reliably measure procedural learning in toddlers in future studies, it may prove to be a useful early screening tool for language difficulties.

Based on our findings, we suggest that language learning might utilise procedural learning or these two might share a common background, and procedural learning holds promise as a potential predictor of language development in children not identified as LTs. To achieve the best results in predicting DLD, we suggest that future studies aim to create a multifactorial model that considers the findings from several studies suggesting multiple predictors for language development. We are likely to need multifactorial models of developmental language trajectories to gain insight into predictors that are not just cumulative, but that interact with each other. One example of such interaction was observed in this study—the different relationship between procedural learning and language outcomes in children with and without a history of LT. LT has been proven to be a significant predictor for later language abilities but without combining it with other predictors, its explanatory power remains modest. Formulating a multifactorial model requires careful assessment of shared variation and mechanisms between different predictors. Thus, these attempts will require combining and investigating the relationships between many predictors: behavioural, experimental, genetic, socio‐economic and cognitive.

## CONFLICTS OF INTEREST

The authors report no conflicts of interest.

## Supporting information

Supplementary material

## Data Availability

Data are available on request due to privacy/ethical restrictions.

## NOTES

1 One child in the control group had no MacArthur Bates Communicative Development Inventory data available but performed within normal limits in the Fox Language Inventory, The Renfrew Word Finding Vocabulary Test, and Reynell Developmental Language Scales III language comprehension at the age of 36 months.

2 Two participants had the hearing level of 30 dB on one ear at 1000 Hz or 4000 Hz in the screening but otherwise passed the screening and had normal hearing according to parent reports.
