# Supplementary material for: Procedural learning and school‐age language outcomes in children with and without a history of late talking
Source: Int J Lang Commun Disord. 2022 Jun 27;57(6):1255–68. doi: 10.1111/1460-6984.12751 (PMC9796386; doi:10.1111/1460-6984.12751)
Supplement: Supplementary file 1 — Supplementary material [file JLCD-57-1255-s001.pdf]

Supporting Information

Procedural learning - sequence learning and motor planning

A. Kautto
5/2022

```
library(ggplot2)
library(lme4)
library(lmerTest)
library(dplyr)
library(ggeffects)
library(sjPlot)
library(jtools)
library(hypr)
library(arsenal)
library(performance)
library(gridExtra)

#set default theme for figures
theme_set(theme_apa()+ theme(legend.position = "bottom"))
```

Data import and correction

```
source("SRTclean.R") #data cleanup script, imports
# 1) df "beh" with behavioral data from 77 participants
# 2) df "data.rt.clean" with filtered Serial Response Time (SRT) RT data (RTs
+/- 2 SD from participant mean, RTs < 100 ms and trials with missing button p
ress / incorrect answer filtered out)
# 3) df "esc" with data from End-State Comfort (ESC) task performance

corrSeq <- read.csv2("corrSeq2.csv")
# see "corrSeq" in sustained attention correction script, includes calculated
values to correct RTs across task phases

data1 <- inner_join(data.rt.clean, corrSeq, by = c("Trial" = "X"))

data1 <- data1 %>%
  mutate(corrRT = RT-x) #corrRT variable includes corrected response times

source("toBlock25.R") #this simple function calculates block numbers from tri
al numbers, trials 1-25 -> 1 etc.
data1$block25 <-  sapply(data1$totaltrial, toblock25)


data1$blockF25 <- relevel(as.factor(data1$block25), "8")
data1$blockF25 <- as.factor(sub("^", "b", data1$blockF25)) # only run once!
```

Serial Response Time (SRT) performance

Hypothesis driven contrast coding

```
mycontr <- hypr(decr = b8~b1, taskeff= b8~(b9+b10+b11+b12)/4, levels =c("b1",
"b10", "b11", "b12", "b2", "b3", "b4", "b5", "b6", "b7", "b8", "b9"))

mdl70 <- lmer(log(RT)~ (blockF25+Lindex.C+riski)^3 + (1|id), data = data1,
              contrasts = list(blockF25 = contr.hypothesis(mycontr)))

#performance::check_model(mdl70) #Model OK
tab_model(mdl70, show.stat = T)
```

	log(RT)			
Predictors	*Estimates*	*CI*	*Statistic*	*p*
(Intercept)	6.42	6.36 – 6.48	213.26	**<0.001**
blockF25decr	-0.11	-0.14 – -0.08	-7.35	**<0.001**
blockF25taskeff	0.05	0.02 – 0.07	3.87	**<0.001**
Lindex.C	-0.11	-0.17 – -0.05	-3.76	**<0.001**
riski [Late talker]	-0.11	-0.19 – -0.03	-2.70	**0.007**
blockF25decr * Lindex.C	-0.04	-0.07 – -0.01	-2.58	**0.010**
blockF25taskeff * Lindex.C	-0.04	-0.06 – -0.01	-2.95	**0.003**
blockF25decr * riski [Late talker]	-0.02	-0.06 – 0.02	-1.04	0.298
blockF25taskeff * riski [Late talker]	-0.02	-0.06 – 0.01	-1.40	0.160
Lindex.C * riski [Late talker]	0.08	-0.00 – 0.16	1.87	0.062
(blockF25decr * Lindex.C) * riski [Late talker]	0.03	-0.02 – 0.07	1.25	0.210
(blockF25taskeff * Lindex.C) * riski [Late talker]	0.06	0.02 – 0.09	3.24	**0.001**

**Random Effects**	
σ^2	0.09
$\tau_{00\ id}$	0.03
ICC	0.25
N_{id}	76
Observations	20047
Marginal R^2 / Conditional R^2	0.071 / 0.305

```
pred70 <- ggpredict(mdl70, terms = c("blockF25[b1, b8, b9]", "Lindex.C[-1.5,
0, 1.5]", "riski"))
srtplot <- plot(pred70, connect.lines = T, use.theme = F) +
  labs(x = "Block of 25 trials", y = "Response time", title = "Predicted valu
es of RTs", color = "") +
    theme(axis.title = element_text(size=14, family = "Times New Roman"),
        legend.title = element_text(size=14, family = "Times New Roman"),
        strip.text = element_text(size=14, family = "Times New Roman"),
        axis.text = element_text(size=14, family = "Times New Roman"),
        plot.title = element_text(size=16, family = "Times New Roman"),
        legend.position = "none") + #legend same for both plots arranged toge
ther later, remove double
  scale_color_grey()

#### sustained attention corrected

mdl70corr <- lmer(log(corrRT)~ (blockF25+Lindex.C+riski)^3 + (1|id), data = d
ata1,
              contrasts = list(blockF25 = contr.hypothesis(mycontr)))

#performance::check_model(mdl70corr) #Model OK
tab_model(mdl70corr, show.stat = T)
```

	**log(corr RT)**			
Predictors	*Estimates*	*CI*	*Statistic*	*p*
(Intercept)	6.29	6.22 – 6.36	181.20	**<0.001**
blockF25decr	-0.33	-0.37 – -0.30	-18.58	**<0.001**
blockF25taskeff	-0.07	-0.09 – -0.04	-4.63	**<0.001**
Lindex.C	-0.13	-0.20 – -0.06	-3.77	**<0.001**

riski [Late talker]	-0.13	-0.22 – -0.03	-2.69	**0.007**
blockF25decr * Lindex.C	-0.08	-0.12 – -0.05	-4.68	**<0.001**
blockF25taskeff * Lindex.C	-0.06	-0.09 – -0.04	-4.52	**<0.001**
blockF25decr * riski [Late talker]	-0.05	-0.10 – 0.00	-1.94	0.053
blockF25taskeff * riski [Late talker]	-0.04	-0.08 – -0.00	-1.99	**0.047**
Lindex.C * riski [Late talker]	0.09	-0.00 – 0.18	1.89	0.059
(blockF25decr * Lindex.C) * riski [Late talker]	0.06	0.01 – 0.11	2.55	**0.011**
(blockF25taskeff * Lindex.C) * riski [Late talker]	0.08	0.04 – 0.12	4.03	**<0.001**
**Random Effects**				
σ^2	0.11			
$\tau_{00\ id}$	0.04			
ICC	0.25			
N_{id}	76			
Observations	20047			
Marginal R^2 / Conditional R^2	0.095 / 0.320			

```
pred70corr <- ggpredict(mdl70corr, terms = c("blockF25[b1, b8, b9]", "Lindex.C[-1.5, 0, 1.5]", "riski"))
srtplotcorr <- plot(pred70corr, connect.lines = T, use.theme = F) +
  labs(x = "Block of 25 trials", y = "Sustained attention corrected\n response time", title = "Predicted values of corrected RTs", color = "Language Index (centered)") +
    theme(axis.title = element_text(size=14, family = "Times New Roman"),
        legend.text = element_text(size=14, family = "Times New Roman"),
        legend.title = element_text(size=14, family = "Times New Roman"),
        strip.text = element_text(size=14, family = "Times New Roman"),
        axis.text = element_text(size=14, family = "Times New Roman"),
        plot.title = element_text(size=16, family = "Times New Roman")) +
```

```
  scale_color_grey()

####

#tab_model(mdl70, mdl70corr, show.stat = T) #table in article file

#grid.arrange(srtplot, srtplotcorr, nrow=2) #figure in article file
```

End-State Comfort (ESC) performance

```
escdata <- merge(esc, beh, by="id") #merge behavioral and ESC task data
escdata <- escdata %>%
  mutate(Lindex.C = scale(Lindex, center = T, scale = T))

escmdl <- glmer(respESC~(as.factor(reqESC) + Lindex.C + riski)^2 + (1|id), da
ta = escdata, family = binomial(link=logit))
#performance::check_model(escmdl) #Model OK
tab_model(escmdl, show.stat = T) #table also in the article file
```

	resp ESC			
Predictors	*Odds Ratios*	*CI*	*Statistic*	*p*
(Intercept)	63.96	25.46 – 160.71	8.85	**<0.001**
reqESC [1]	0.01	0.00 – 0.02	-10.64	**<0.001**
Lindex.C	0.92	0.41 – 2.07	-0.20	0.843
riski	2.29	0.55 – 9.52	1.14	0.256
reqESC [1] * Lindex.C	0.90	0.46 – 1.76	-0.31	0.755
reqESC [1] * riski	0.28	0.07 – 1.10	-1.82	0.069
Lindex.C * riski	1.41	0.64 – 3.09	0.86	0.392
**Random Effects**				
σ^2	3.29			
$\tau_{00\ id}$	2.05			
ICC	0.38			
N_{id}	77			
Observations	1232			
Marginal R^2 / Conditional R^2	0.567 / 0.733			

```
escpred <- ggpredict(escmdl, terms = c("reqESC", "riski"))
plot(escpred, use.theme = F) +
  scale_color_grey() +
  scale_x_discrete(name = "Control trial                              Target
trial") +
  labs(color = "History of late talking", title = "Predicted probabilities of
comfortable end-state grasps") +
  theme(legend.title = element_text(size=12))
```

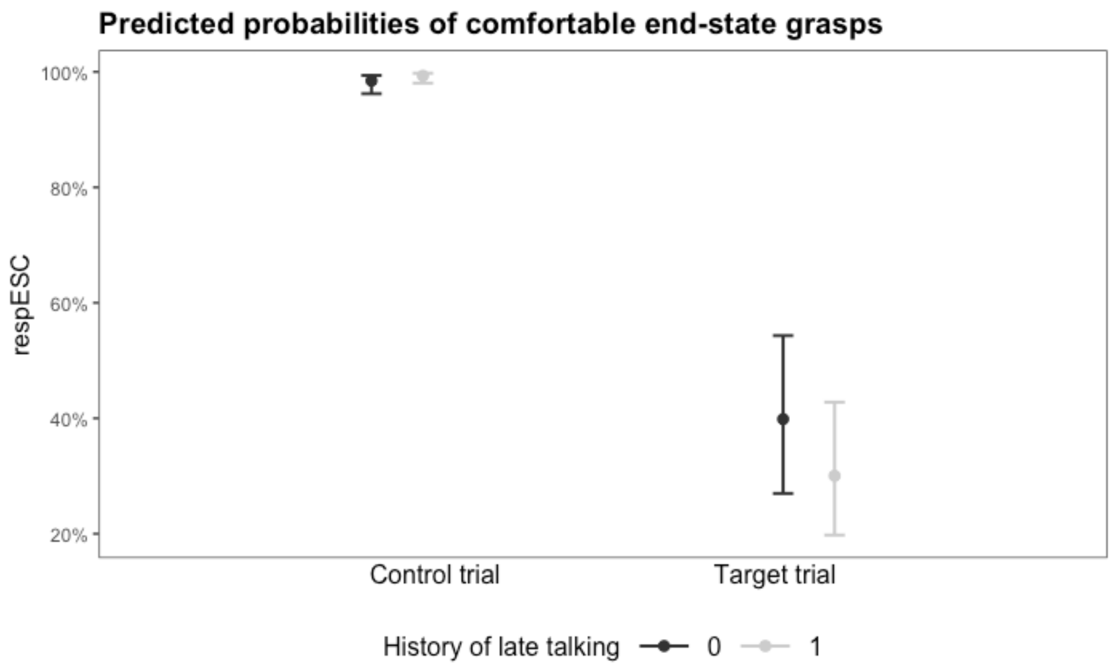

Sustained attention correction to Serial Response Time (SRT) task

A. Kautto
5/2022

Data frames in this script:

1) df “beh” with behavioral data from 77 participants
2) df “data.rt.clean” with filtered SRT RT data (RTs +/- 2 SD from participant mean, RTs < 100 ms and trials with missing button press / incorrect answer filtered out)
3) df “esc” with data from ESC task performance

Libraries used: ggplot2 ggeffects dplyr lme4 gridExtra jtools sjPlot

Sustained attention effect masks learning (as reflected by RT decrease) during SRT task

Learning during the SRT task is reflected by RT decreases. There is also an effect of sustained attention that affects RTs. While learning decreases RTs during task, sustained attention increases them. This results in masked learning effects. Next we plot the modeled response times (before correction) during SRT task. During data collection, the phases were presented in this order (pattern 1, pattern 2, random) and the participants have had a small break between the phases. Note the decrease in response times after the breaks.

```
trialmdl <-  glmer(RT ~ Trial*proc + (Trial|id), data = data.rt.clean,
                   family = inverse.gaussian(link = "identity"),
                   control = glmerControl(optimizer = "bobyqa", optCtrl = lis
t(maxfun=100000)))
tab_model(trialmdl, show.est = F, show.stat = T)
```

	RT	
Predictors	*Statistic*	*p*
(Intercept)	119.21	**<0.001**
Trial	1.72	0.086
proc [PatternPhase2]	-24.07	**<0.001**
proc [Random2]	-23.29	**<0.001**
Trial * proc [PatternPhase2]	6.85	**<0.001**

Trial * proc [Random2]	8.65	**<0.001**

**Random Effects**	
σ^2	0.01
$\tau_{00\ id}$	1519.70
$\tau_{11\ id.Trial}$	0.15
$\rho_{01\ id}$	-0.22
ICC	1.00
N id	76
Observations	20047
Marginal R^2 / Conditional R^2	0.553 / 1.000

```r
# extract estimates

trialpred <- ggpredict(trialmdl, terms=c("Trial [1:100]", "proc"))

# plot model

predplot <- ggplot(trialpred, aes(x=x, y=predicted))+
  geom_point(position=position_dodge(0.1), size=.1) +
  geom_ribbon(aes(ymin=conf.low, ymax=conf.high), alpha = 0.3) +
  xlab("Trial number") +
  ylab("Response time (ms)") +
  facet_wrap(~group) +
  theme(axis.title = element_text(size=14, family = "Times New Roman"),
        legend.text = element_text(size=14, family = "Times New Roman"),
        legend.title = element_text(size=14, family = "Times New Roman"),
        strip.text = element_text(size=14, family = "Times New Roman"),
        axis.text = element_text(size=14, family = "Times New Roman"),
        plot.title = element_text(size=16, family = "Times New Roman"))
predplot
```

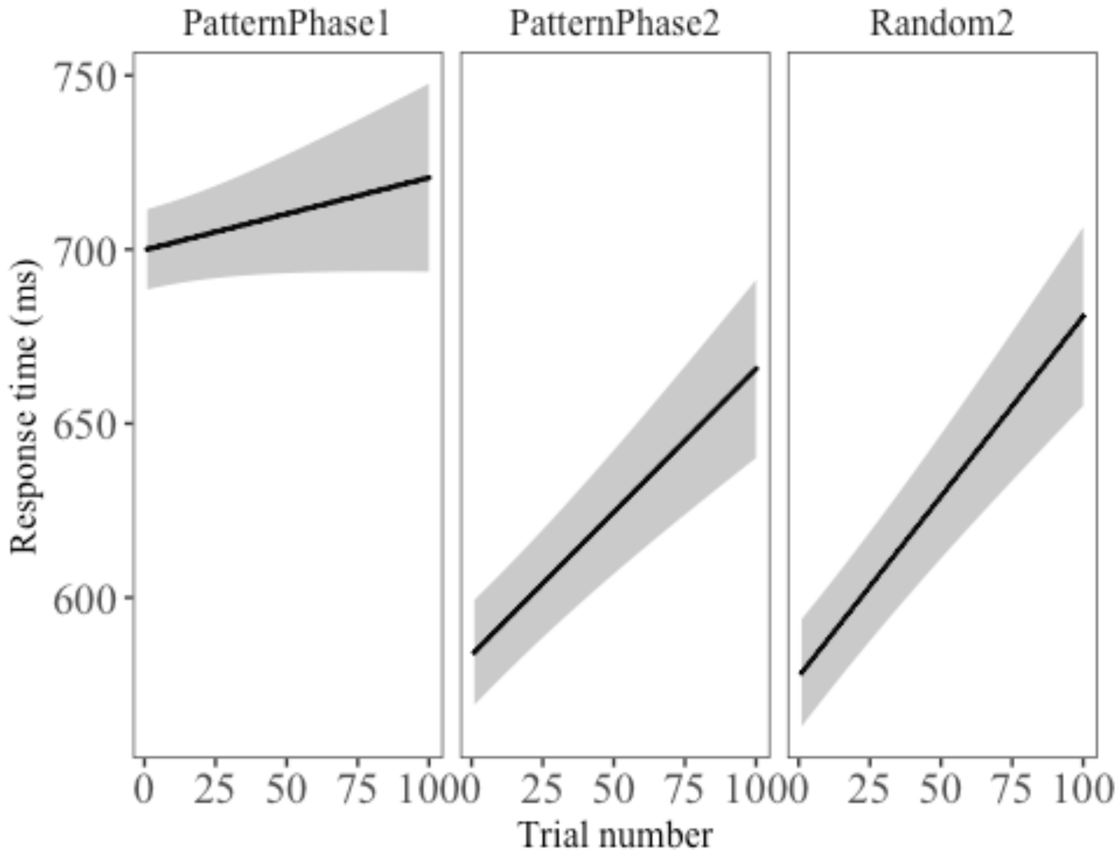

Assumptions:

- The effect of sustained attention decrease is linear. This means the effect increases linearly from reference level (adjusted to zero) to task maximum (end of the block).
- Best estimate for sustained attention decrease maximum is the difference between pattern 1 trial 100 and pattern 2 trial 1 estimate. We consider this to be the explanation for this difference since pattern 1 and pattern 2 blocks are similar (consolidation during rest possible but its effect is not likely very big).
- Effect of sustained attention is similar in all blocks (pattern 1 / pattern 2 / random) While learning varies as a function of block type and prior learning, sustained attention decrease across blocks is similar. This is a rough estimate; there is likely slight differences associated with task demands (likely steeper increase in RTs in a demanding task) and block order (despite resting in between blocks, later blocks might be more affected by sustained attention). However, in our data the participant could adjust the length of rest period between the trials and continue when they felt “ready”.

```
# calculate corrected values for RTs
  # calculate values to be substracted (linearly from 0 to (seq1trial100-seq2
trial1))
  # & create evenly spaced double values sequence: 100 data points [0, seq1tr
ial100-seq2trial1]
```

```
corrSeq <- seq(from=0, to=(trialpred[298,2]-trialpred[2,2]), length.out = 100
)

# new df to avoid mess in plotting

trialpred2 <- trialpred

# sequence to data frame as a new column "corrVal"
for (i in 1:nrow(trialpred2)) {
  trial <- trialpred2$x[i]
  trialpred2$corrVal[i] <- corrSeq[trialpred2$x[i]]
}


trialpred2 <- trialpred2 %>%
  mutate(fatiqCorr = predicted-corrVal) %>%
  mutate(conf.lowC = conf.low-corrVal) %>%
  mutate(conf.highC = conf.high-corrVal)


predplotC <- ggplot(trialpred2, aes(x=x, y=fatiqCorr))+
  geom_point(position=position_dodge(0.1), size=.1) +
  geom_ribbon(aes(ymin=conf.lowC, ymax=conf.highC), alpha = 0.3) +
  xlab("Trial number") +
  ylab("Sustained attention corrected\n response time (ms)") +
  facet_wrap(~group) +
  theme(axis.title = element_text(size=14, family = "Times New Roman"),
        legend.text = element_text(size=14, family = "Times New Roman"),
        legend.title = element_text(size=14, family = "Times New Roman"),
        strip.text = element_text(size=14, family = "Times New Roman"),
        axis.text = element_text(size=14, family = "Times New Roman"),
        plot.title = element_text(size=16, family = "Times New Roman"))

grid.arrange(predplot, predplotC, nrow =2)
```

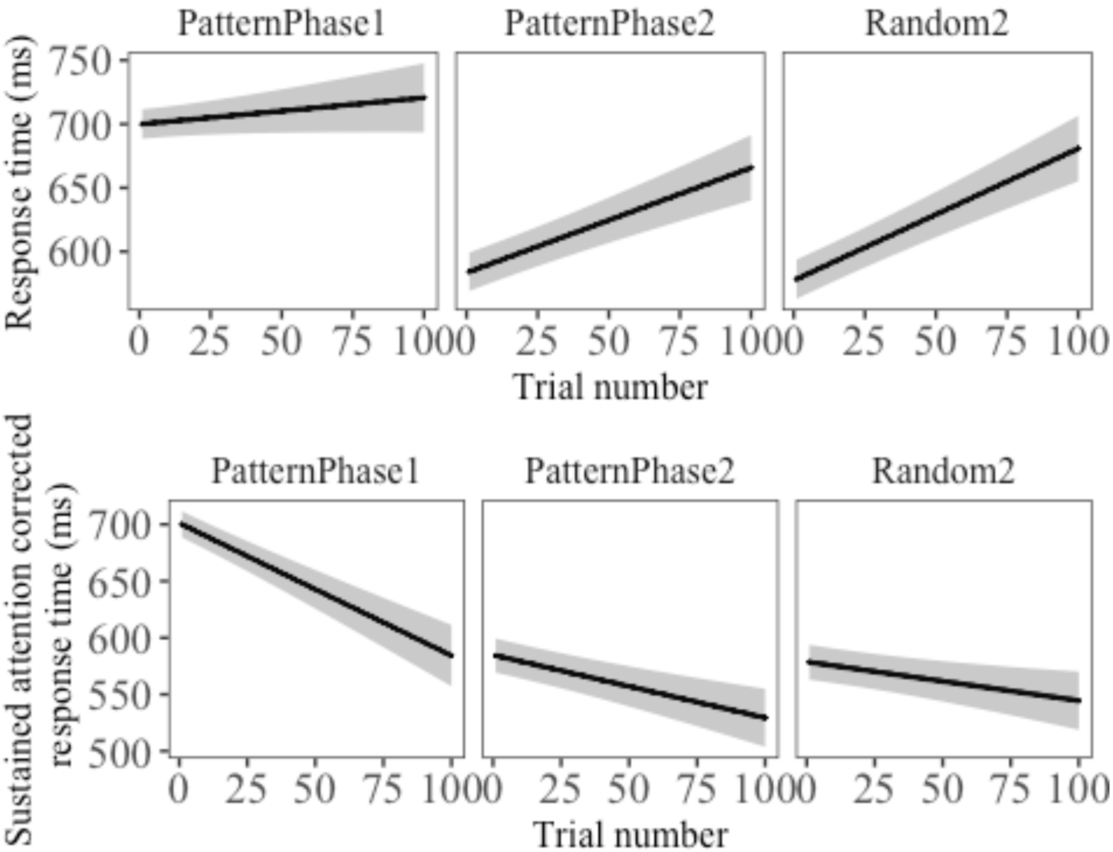

RTs before sustained attention correction (above) and after it (below). The correction reveals SRT task effect: RTs increase from pattern blocks to random. There seems to be some learning during random procedure, as reflected by RT decreases. We propose that the size of sustained attention effect in pattern 2 and random blocks is underestimated rather than overestimated since it is based on pattern 1. However, we could not safely assume the effect to be bigger than that.

Apparatus used in the dowel task

Example instruction: ”Place the black end of the dowel in cup two.”

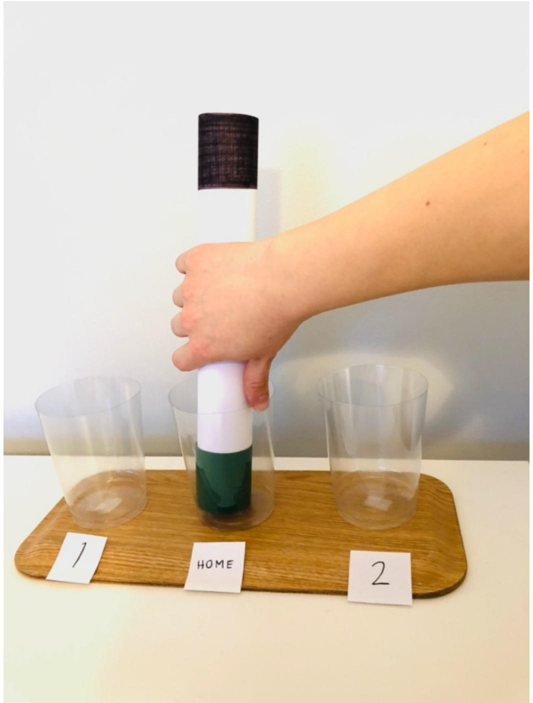

1) Uncomfortable initial grasp…

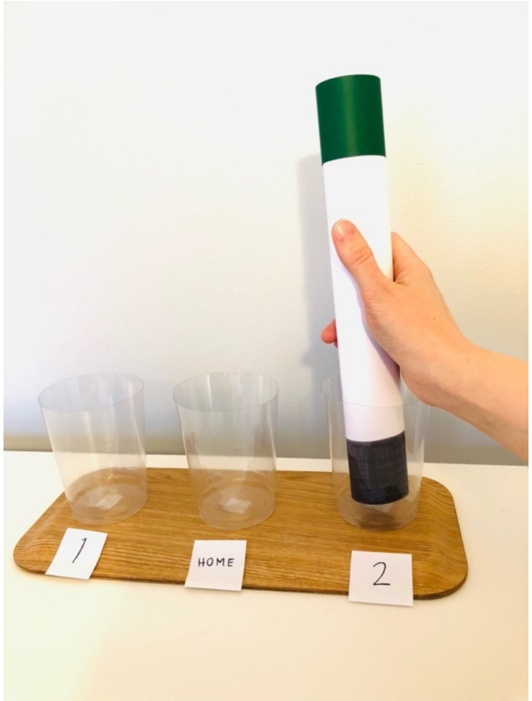

2) …leads to comfortable end state grasp.
